# Identification and characterization of two P450 enzymes from *Citrus sinensis* involved in TMTT and DMNT biosyntheses and Asian citrus psyllid defense

**DOI:** 10.1093/hr/uhae037

**Published:** 2024-04-01

**Authors:** Xueli Sun, Chunhua Hu, Ganjun Yi, Xinxin Zhang

**Affiliations:** Key Laboratory of South Subtropical Fruit Biology and Genetic Resource Utilization, Ministry of Agriculture and Rural Affairs; Guangdong Provincial Key Laboratory of Tropical and Subtropical Fruit Tree Research, Institute of Fruit Tree Research, Guangdong Academy of Agricultural Sciences, Guangzhou 510640, China; College of Life Sciences, State Key Laboratory for Conservation and Utilization of Subtropical Agro-bioresources, South China Agricultural University, Guangzhou 510642, China; Key Laboratory of South Subtropical Fruit Biology and Genetic Resource Utilization, Ministry of Agriculture and Rural Affairs; Guangdong Provincial Key Laboratory of Tropical and Subtropical Fruit Tree Research, Institute of Fruit Tree Research, Guangdong Academy of Agricultural Sciences, Guangzhou 510640, China; Key Laboratory of South Subtropical Fruit Biology and Genetic Resource Utilization, Ministry of Agriculture and Rural Affairs; Guangdong Provincial Key Laboratory of Tropical and Subtropical Fruit Tree Research, Institute of Fruit Tree Research, Guangdong Academy of Agricultural Sciences, Guangzhou 510640, China; Key Laboratory of South Subtropical Fruit Biology and Genetic Resource Utilization, Ministry of Agriculture and Rural Affairs; Guangdong Provincial Key Laboratory of Tropical and Subtropical Fruit Tree Research, Institute of Fruit Tree Research, Guangdong Academy of Agricultural Sciences, Guangzhou 510640, China

## Abstract

The homoterpenes (3*E*)-4,8-dimethyl-1,3,7-nonatriene (DMNT) and (*E*,*E*)-4,8,12-trimethyl-1,3,7,11-tridecatetraene (TMTT) are the major herbivore-induced plant volatiles that help in defense directly by acting as repellants and indirectly by recruiting insects’ natural enemies. In this study, DMNT and TMTT were confirmed to be emitted from citrus (*Citrus sinensis*) leaves infested with Asian citrus psyllid (*Diaphorina citri* Kuwayama; ACP), and two cytochrome P450 (CYP) genes (*CsCYP82L1* and *CsCYP82L2*) were newly identified and characterized. Understanding the functions of these genes in citrus defense will help plan strategies to manage huanglongbing caused by *Candidatus* Liberibacter asiaticus (*C*Las) and spread by ACP. Quantitative real-time PCR (qPCR) analysis showed that *CsCYP82L1* and *CsCYP82L2* were significantly upregulated in citrus leaves after ACP infestation. Yeast recombinant expression and enzyme assays indicated that CsCYP82L1 and CsCYP82L2 convert (*E*)-nerolidol to DMNT and (*E,E*)-geranyllinalool to TMTT. However, citrus calluses stably overexpressing *CsCYP82L1* generated only DMNT, whereas those overexpressing *CsCYP82L2* produced DMNT and TMTT. Furthermore, ACPs preferred wild-type lemon (*Citrus limon*) over the *CsCYP82L1*-overexpressing line in dual-choice feeding assays and mineral oil over TMTT or DMNT in behavioral bioassays. Finally, yeast one-hybrid, electrophoretic mobility shift, and dual luciferase assays demonstrated that CsERF017, an AP2/ERF transcription factor, directly bound to the CCGAC motif and activated *CsCYP82L1*. Moreover, the transient overexpression of *CsERF017* in lemon leaves upregulated *CsCYP82L1* in the absence and presence of ACP infestation. These results provide novel insights into homoterpene biosynthesis in *C. sinensis* and demonstrate the effect of homoterpenes on ACP behavior, laying a foundation to genetically manipulate homoterpene biosynthesis for application in huanglongbing and ACP control.

## Introduction

Plants have evolved complex defense mechanisms against fungi, viruses, bacteria, nematodes, insects, and other herbivores [1]. They produce and accumulate various defense-related metabolites in response to the attacks by these organisms [2]. Among the various metabolites, herbivore-induced plant volatiles (HIPVs) aid in host defense by acting directly against the herbivores or by attracting herbivores’ natural enemies [3].

Progress in analytical techniques has led to identifying diverse volatiles with significant roles in plant protection [4–6]. Among the various volatiles, (*E*,*E*)-4,8,12-trimethyltrideca-1,3,7,11-tetraene (TMTT), a C_16_ homoterpene, and (*E*)-4,8-dimethyl-1,3,7-nonatriene (DMNT), the C_11_ analog, have been identified as the predominant ones emitted by plants following herbivory [7–9]. Both of these irregular acyclic terpenes are common floral scent compounds [10]. Like all HIPVs, TMTT and DMNT are known to act directly or indirectly on herbivores. In *Arabidopsis thaliana*, TMTT released after *Pieris rapae* attack attracts the parasitoid wasp *Cotesia rubecula*, which is a natural enemy of the pest [11]. Similarly, cotton (*Gossypium hirsutum*) infested by the bollworm (*Helicoverpa armigera*) and the mirid bug *Apolygus lucorum* releases both TMTT and DMNT, which attract their natural enemies, *Microplitis mediator* and *Peristenus spretus* [12]. On the other hand, TMTT in maize is known to strongly repel the streak virus vector, *Cicadulina storeyi*, whereas both TMTT and DMNT in potato (*Solanum tuberosum* L.) repel the aphid *Macrosiphum euphorbiae* [13, 14]. Besides, Jing *et al*. [15] showed that jasmonic acid (JA) application in tea (*Camellia sinensis*) induces the emission of DMNT, which enhances the resistance of neighboring tea plants to herbivore attack. These earlier studies demonstrated the crucial roles of TMTT and DMNT in protecting plants from insects and attracting the natural enemies of insect pests. However, reports on the role of these HIPVs in citrus are rare.

Citrus is a fruit crop widely grown and consumed around the world. One of the most devastating diseases in citrus is huanglongbing (HLB), caused by the bacterial species *Candidatus* Liberibacter asiaticus (*C*Las). The Asian citrus psyllid (ACP) *Diaphorina citri* is known to transmit the HLB-associated bacterial species *C*Las [16]. However, currently there is no effective measure to control HLB. Previous studies on the volatiles of a few horticultural crops demonstrated that *Anacardium occidentale* contained high levels of DMNT and TMTT, which were not preferred by ACP in behavioral assays [17]. This observation suggested that the homoterpenes DMNT and TMTT do not attract ACP. Therefore, using semiochemicals, such as DMNT and TMTT, to manage the behavior of the bacterial carrier or vector spreading HLB seems like a promising strategy to protect citrus plants.

**Figure 1:**
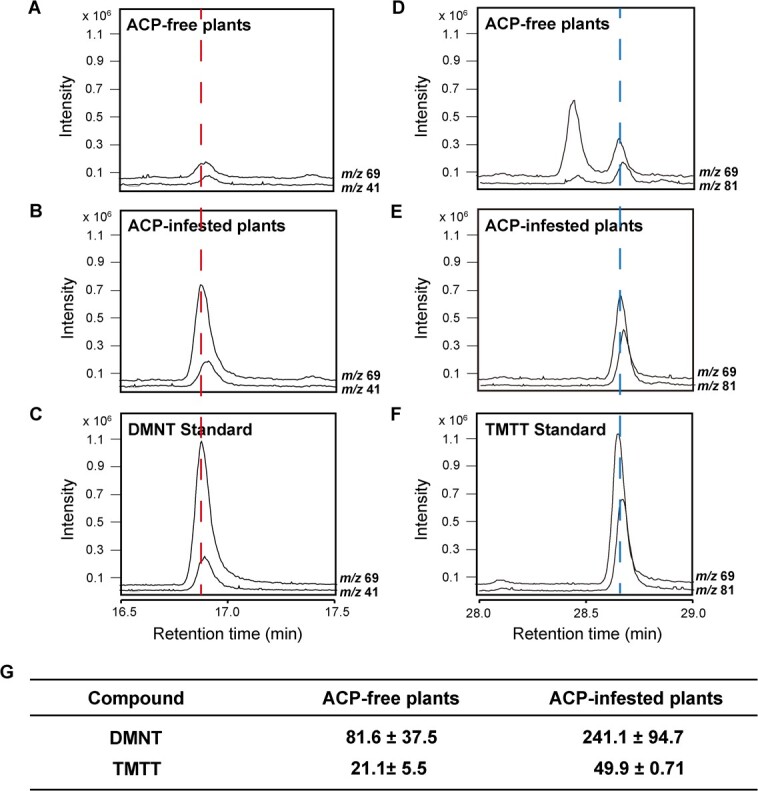
Qualitative and quantitative analyses of DMNT and TMTT emitted by citrus plants. **A**–**C** Qualitative analysis of DMNT emitted by citrus before and after ACP infestation. Here, *m*/*z* 41 and 69 represent the characteristic ion mass charge ratios of DMNT. **D**–**F** Qualitative analysis of TMTT emitted from citrus before and after ACP infestation. Here, *m*/*z* 69 and 81 represent the characteristic ion mass charge ratios of TMTT. The red dashed line indicates the characteristic ion intensity peak of DMNT, and the blue dashed line indicates the characteristic ion intensity peak of TMTT. **G** Quantitative analysis of DMNT and TMTT emitted by the control and infested citrus. Data (means ± standard deviation) are expressed in ng/h.

Research has demonstrated that AtCYP82G1, a cytochrome P450 (CYP) isolated from *A. thaliana*, participates in TMTT and DMNT biosynthetic pathways, using (*E*,*E*)-geranyllinalool and (*E*)-nerolidol as the substrates [9, 18]. However, the role of the *CYP* genes in regulating the synthesis of TMTT and DMNT in citrus and the influence of these compounds on male or female *D. citri* remain unclear. Recently, we found that ACP infestation upregulated two *CYP* genes of *C. sinensis*, *CsCYP82L1* and *CsCYP82L2* [19]. Nevertheless, their functions in the citrus defense system have not been explored.

Therefore, the present study explored the components of the TMTT and DMNT biosynthetic pathways and analyzed their exact functions in citrus. We first analyzed DMNT and TMTT emissions from ACP-infested citrus plants by gas chromatography–mass spectrometry (GC–MS) and determined the transcript abundance of *CsCYP82L1* and *CsCYP82L2* in the citrus tissues after ACP infestation and in response to mechanical damage and phytohormone treatment by quantitative real-time PCR (qPCR). We further measured CsCYP82L1 and CsCYP82L2 activities after expression in yeast (*Saccharomyces cerevisiae*) and citrus calluses by applying solid-phase microextraction (SPME) and GC–MS and validated their roles in homoterpene metabolism. Furthermore, to explore the functions, we examined the preference of ACPs for the *CsCYP82L1-*overexpressing plant and the wild-type (WT) plant and the influence of pure TMTT and DMNT on ACPs in a behavioral bioassay using a Y-tube olfactometer. Finally, we investigated the role of a transcription factor (CsERF017) in activating *CsCYP82L1* using the yeast one-hybrid assay (Y1H), electrophoretic mobility shift assay (EMSA), tobacco transient dual luciferase assay, and lemon leaf transient expression.

## Results

### DMNT and TMTT emission from ACP-infested citrus

Headspace gas analysis using GC–MS showed that ACP-infested citrus plants emitted more DMNT and TMTT than the ACP-plants (Fig. 1A, B, D, E), indicating that ACP infestation could strongly induce DMNT and TMTT release. The amounts of DMNT and TMTT produced were calculated by comparing the peak area ratio to an internal standard (Fig. 1G). These observations confirmed the biosynthesis of homoterpenes in citrus and the involvement of specific genes in the associated metabolic pathway.

### Identification of *CsCYP* genes participating in TMTT and DMNT biosyntheses

**Figure 2:**
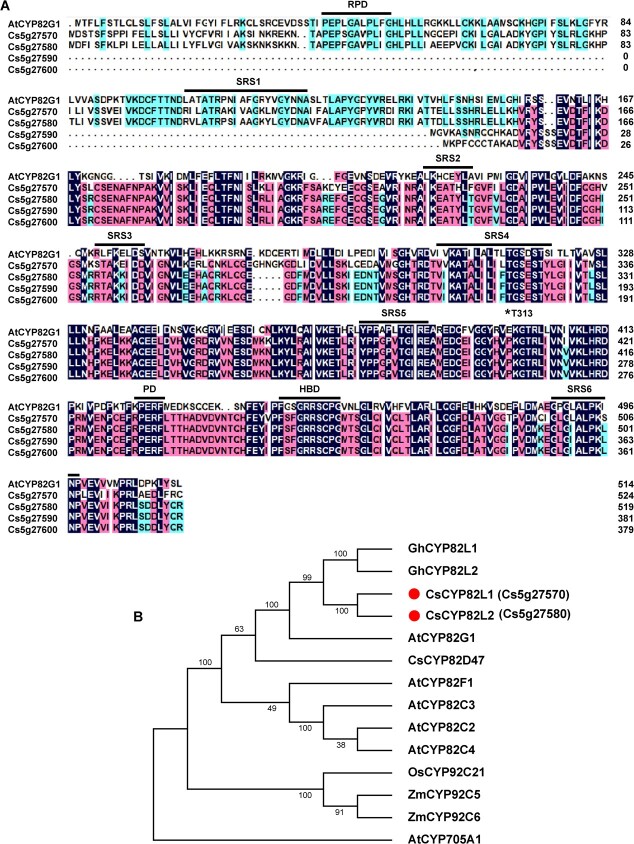
Amino acid alignment and phylogenetic analysis of CsCYPs with other plant CYPs. **A** Amino acid alignment of the candidate CsCYPs of *C. sinensis* with AtCYP82G1 of *A. thaliana*. PD, PRD, and HBD indicate PERF, proline-rich, and heme-binding domains; SRS indicates the substrate recognition sites, and SRS1–SRS6 represent the SRSs of AtCYP82G1. Amino acids with asterisks are the key substrate-interacting residues identified in AtCYP82G1. **B** Phylogeny based on the CYP sequences from *A. thaliana* (AtCYP82C2, At4g31970; AtCYP82C3, At4g31950; AtCYP82C4, At4g31940; AtCYP82F1, At2g25160; AtCYP82G1, At3g25180; AtCYP705A1, At4g15330), *C. sinensis* (CsCYP82D47, THG22758.1), *C. sinensis* (CsCYP82L1, Cs5g27570; CsCYP82L2, Cs5g27580), *G. hirsutum* (GhCYP82L1, KY247144.1; GhCYP82L2, KY247145.1), *O. sativa* (OsCYP92C21, ABF97912.1), and *Z. mays* (ZmCYP92C5, ACG28049.1; ZmCYP92C6, GRMZM2G139467). ClustalW performed the multiple sequence alignment, and MEGA-X built the phylogenetic tree. Here, the sequences marked with red dots represent the candidate CYPs of *C. sinensis* associated with homoterpene synthesis. Bootstrap values are shown as percentages based on 1000 bootstrap replicates at the nodes.

TMTT and DMNT are volatiles essential for triggering herbivory-induced responses. In various plant species, P450s are known to catalyze the final degradation step during homoterpene formation [9]. However, the genes encoding the enzymes involved in catalyzing TMTT and DMNT syntheses in citrus remain undetermined. In this study, detecting DMNT and TMTT released from the citrus plants after ACP infestation (Fig. 1) prompted us to identify the genes involved in their biosyntheses. Therefore, we first used the amino acid sequence of *AtCYP82G1*, associated with TMTT production in *Arabidopsis*, and searched for putative candidates in the *C. sinensis* reference genome (v2.1; http://citrus.hzau.edu.cn/) [20]. Based on the conserved domains of the P450s [PERF (PD), proline-rich (PRD), and heme-binding (HBD) domains] and the substrate recognition sites (SRSs) of AtCYP82G1 (Fig. 2A), the genes with the locus identifier numbers *Cs5g27570*, *Cs5g27580*, *Cs5g27590*, and *Cs5g27600* were selected as *CsCYP* candidate genes.

Further, we obtained the expression profiles of four P450 genes in citrus leaves after ACP infestation using published RNA-seq data to identify the significant ones [19]. Interestingly, ACP infestation downregulated *Cs5g27590* and *Cs5g27600* compared with the control but upregulated *Cs5g27570* and *Cs5g27580* (Supplementary Data Table S1). Subsequently, we built a phylogenetic tree using the sequences of Cs5g27570, Cs5g27580, and the known P450 proteins from *A. thaliana*, *C. sinensis*, *G. hirsutum*, *Oryza sativa*, and *Zea mays* (Fig. 2B). The analysis revealed an 84.76% sequence identity between Cs5g27570 and Cs5g27580, and Cs5g27580 showed the highest identity with GhCYP82L2 (62.67%) and GhCYP82L1 (61.64%) from *G. hirsutum* (Fig. 2B). The names of the two GhCYP82Ls were obtained based on the reported nomenclature and phylogenetic classification of the P450s [12]. Thus, the present study grouped Cs5g27570 and Cs5g27580 into the CYP82L family and named them CsCYP82L1 and CsCYP82L2, respectively.

### Expression of *CsCYP82L1* and *CsCYP82L2* after ACP infestation and in response to mechanical damage and phytohormone treatment

**Figure 3:**
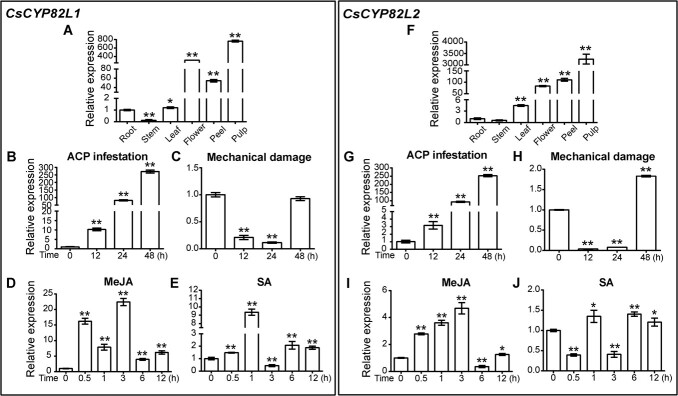
Expression of *CsCYP82L1* and *CsCYP82L2* in different tissues (A, F) under normal conditions and leaves in response to ACP infestation (B, G), mechanical damage (C, H), and phytohormone (MeJA, SA) treatment (D, I; E, J) of *C. sinensis*. **A**, **F** Expression of *CsCYP82L1* and *CsCYP82L2* in different tissues. **B**, **G** Expression of *CsCYP82L1* and *CsCYP82L2* after ACP infestation. **C**, **H** Expression of *CsCYP82L1* and *CsCYP82L2* after mechanical damage. **D**, **I** Expression of *CsCYP82L1* and *CsCYP82L2* after MeJA treatment. **E**, **J** Expression of *CsCYP82L1* and *CsCYP82L2* after SA treatment. Expression levels of *CsCYP82L1* and *CsCYP82L2* were measured relative to the housekeeping gene *CsActb*. Data shown are mean values ± standard deviation of three independent experiments. In **A** and **F**, * and ** indicate significant differences between root and other tissues at *P* < 0.05 and *P* < 0.01, respectively. In **B**–**E** and **G**–**J**, * and ** indicate significant differences compared with 0 h at *P* < 0.05 and *P* < 0.01, respectively.

To further explore the functions of the two *CsCYP82Ls*, we first examined their relative expression levels in different citrus tissues. *CsCYP82L1* and *CsCYP82L2* were highly expressed in flower, peel, and pulp (Fig. 3A and F). Besides, consistent with the observations based on RNA-seq data (Supplementary Data Table S1), qRT–PCR demonstrated higher *CsCYP82L1* and *CsCYP82L2* expression in ACP-infested plants than in control plants (Fig. 3B and G). At 48 h after ACP infestation, *CsCYP82L1* and *CsCYP82L2* showed a 273- and 253-fold increase in expression compared with control (Fig. 3B and G). However, mechanical damage suppressed *CsCYP82L1* and *CsCYP82L2* expression in citrus at 12 and 24 h compared with the 0 h control; here, *CsCYP82L2* alone was slightly upregulated at 48 h (Fig. 3C and H).

Phytohormones, especially jasmonic acid (JA) and salicylic acid (SA), have been associated with the response mechanisms of plants following herbivore and pathogen attacks [21–23]. Studies have demonstrated that JA mediates host resistance to various insects via a mechanism conserved across species. The JA-related response involves altering transcript levels, stimulating defense proteins and peptides, and forming secondary metabolites [21, 24]. To examine the role of *CsCYP82L1* and *CsCYP82L2* in insect resistance-related signaling, we applied methyl jasmonate (MeJA) and SA exogenously to citrus plants and analyzed the levels of *CsCYP82L1* and *CsCYP82L2* transcripts. Our analysis showed that *CsCYP82L1* and *CsCYP82L2* were upregulated immediately after MeJA treatment (0.5 h) and stayed at high levels even at 3 h (Fig. 3D and I). SA treatment also quickly upregulated *CsCYP82L1* expression (0.5 h) but downregulated *CsCYP82L2* expression (Fig. 3E and J). These observations indicated a correlation between the *CsCYP82L*s and insect resistance-associated signaling pathways in *C. sinensis*.

### Subcellular localization of CsCYP82L1 and CsCYP82L2

**Figure 4:**
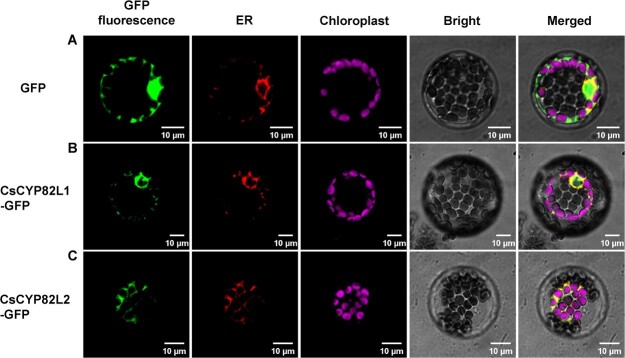
Subcellular localization of CsCYP82L1 and CsCYP82L2. Signals of CsCYP82L1/CsCYP82L2-GFP were detected on the ER membrane of *Arabidopsis* protoplasts. 35S:CsCYP82L1-GFP or 35S:CsCYP82L2-GFP were introduced with pCAMBIA1300-sper-mKATE containing an ER membrane-localization signal peptide into protoplasts isolated from the leaves of *Arabidopsis*; 35S:GFP was used as the control. The fluorescence was visualized by confocal laser microscopy. Scale bars = 10 μm.

Protein localization in a cell helps predict its *in vivo* functions. In this study, we introduced the green fluorescent protein (GFP) fusion constructs of the two CYPs separately into protoplasts obtained from *Arabidopsis* mesophyll cells, using an empty GFP vector as a control, and analyzed the location. The signal due to the empty GFP vector was detected in the cytosol and the nucleus (Fig. 4A). In contrast, CsCYP82L1 and CsCYP82L2 fused with GFP were co-localized with the endoplasmic reticulum (ER) membrane-localization signal peptide (Fig. 4B and C). These data suggested that CsCYP82L1 and CsCYP82L2 are mainly localized to the ER membrane.

### *In vitro* activity of CsCYP82L1 and CsCYP82L2 enzymes

**Figure 5:**
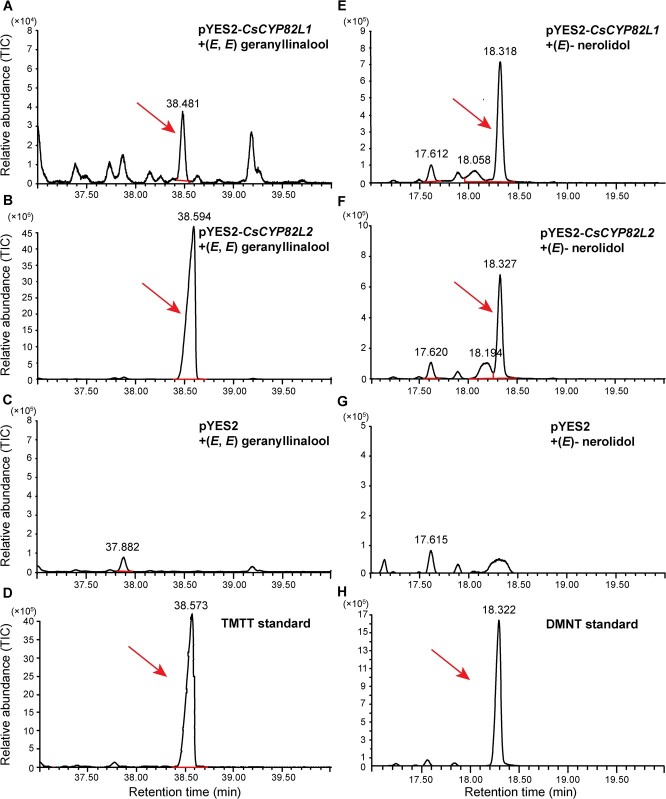
GC–MS of TMTT and DMNT synthesized in *CsCYP82L1* or *CsCYP82L2*-expressing yeast cells in the presence of exogenously added substrate (*E*,*E*)-geranyllinalool or (*E*)-nerolidol. A gas chromatograph coupled with a mass spectrometer was used to analyze the products. **A** TMTT produced by recombinant CsCYP82L1. **B** TMTT produced by recombinant CsCYP82L2. **C** Volatiles produced by yeast cells carrying empty vector pYES2. **D** Gas chromatogram of the TMTT authentic standard. **E** DMNT formed by recombinant CsCYP82L1. **F** DMNT produced by recombinant CsCYP82L2. **G** Volatiles produced by yeast cells carrying empty vector pYES2. **H** Gas chromatogram of the DMNT authentic standard.

To further verify whether CsCYP82L1 and CsCYP82L2 participate in synthesizing TMTT and DMNT, we assessed the activity of CsCYP82L1 and CsCYP82L2 enzymes in yeast cells with exogenously added (*E*,*E*)-geranyllinalool or (*E*)-nerolidol as the substrate (Fig. 5). GC–MS detected both TMTT and DMNT as the products of CsCYP82L1 or CsCYP82L2 enzymatic activity based on the mass spectra (MS) and the retention time of the authentic standards (Fig. 5A, B, E, and F). However, neither TMTT nor DMNT was detected within the yeast cells carrying the empty expression vector pYES2 (Fig. 5C and G). These results implied that the recombinant CsCYP82L1 and CsCYP82L2 could convert (*E*,*E*)-geranyllinalool to TMTT or (*E*)-nerolidol to DMNT.

### *In vivo* activity of CsCYP82L1 and CsCYP82L2 enzymes

**Figure 6:**
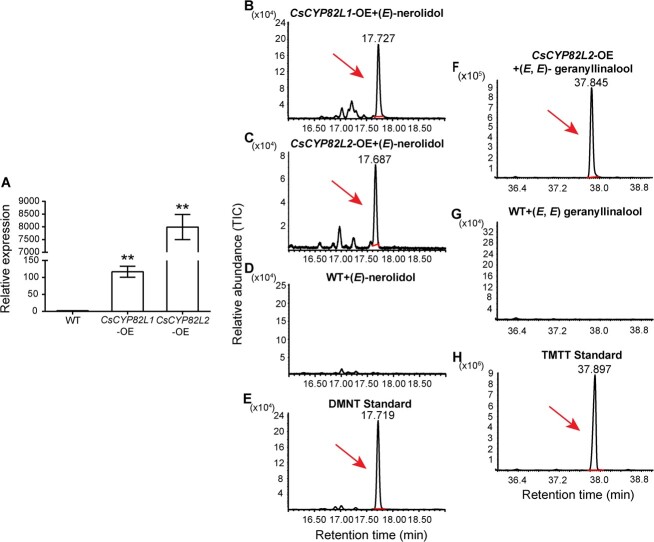
Activity of CsCYP82L1 and CsCYP82L2 enzymes after overexpression of their genes in transgenic citrus callus. **A** Positive transgenic citrus callus was identified by examining relative expression levels of *CsCYP82L1* and *CsCYP82L2* using qRT–PCR analysis. Data are presented as mean values ± standard error (*n* = 3). Here, ** indicates significant differences between WT and transgenic plants at *P* < 0.01. **B**–**H** GC–MS analysis of TMTT and DMNT produced by citrus callus overexpressing *CsCYP82L1* or *CsCYP82L2* using the substrate (*E*,*E*)-geranyllinalool or (*E*)-nerolidol. **B** DMNT produced by *CsCYP82L1*-OE citrus callus, **C** DMNT produced by *CsCYP82L2*-OE citrus callus, **D** volatiles produced by WT citrus callus, **E** GC–MS analysis of authentic DMNT standard, **F** TMTT produced by *CsCYP82L2*-OE citrus callus, **G** volatiles produced by WT citrus callus, and **H** GC–MS analysis of authentic TMTT standard.

Then, to verify the enzymatic activity of CsCYP82L1 and CsCYP82L2 *in vivo*, we overexpressed *CsCYP82L1* and *CsCYP82L2* in citrus callus and confirmed their levels compared with control via qRT–PCR (Fig. 6A). Subsequent GC–MS analysis showed that WT citrus callus lacked the peaks of TMTT or DMNT, indicating that WT citrus callus could not produce detectable levels of TMTT or DMNT using exogenously added (*E*,*E*)-geranyllinalool or (*E*)-nerolidol substrate (Fig. 6D and G). The *CsCYP82L1-*OE callus produced DMNT alone, while the *CsCYP82L2-*OE callus produced DMNT using the substrate (*E*)-nerolidol and TMTT using the substrate (*E*,*E*)-geranyllinalool (Fig. 6B, C, and F). These results revealed that CsCYP82L1 could form DMNT from (*E*)-nerolidol, and CsCYP82L2 could form TMTT from (*E*,*E*)-geranyllinalool and DMNT from (*E*)-nerolidol in citrus callus.

### Transgenic lemon overexpressing *CsCYP82L1* demonstrates decreased susceptibility to ACP primarily due to DMNT and TMTT

**Figure 7:**
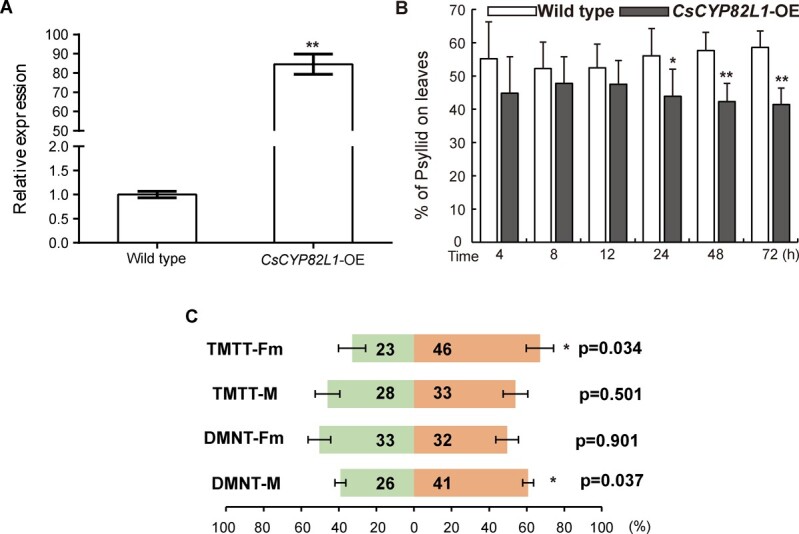
Preference of ACP for WT and *CsCYP82L1-*overexpressing lemon plants and behavioral response of ACP to DMNT and TMTT. **A** Identification of transgenic lemon plants overexpressing *CsCYP82L1* using qRT–PCR. Expression of *CsCYP82L1* was examined in WT and transgenic lemon plants. Here, data are presented as mean values ± standard error (*n* = 3); ** indicates significant differences between WT and transgenic plants at *P* < 0.01. **B** Overexpression of *CsCYP82L1* in lemon influences ACP feeding preference. Data shown are mean values ± standard error (*n* = 5). Here, * and ** indicate significant differences between WT and transgenic plants at *P* < 0.05 and *P* < 0.01, respectively. **C** Behavioral responses of male and female ACPs to TMTT and DMNT. DMNT-Fm, female ACPs that preferred mineral oil (orange bars) over DMNT (green); DMNT-M, male ACPs that preferred mineral oil (orange bars) over DMNT (green bars); TMTT-Fm, female ACPs that preferred mineral oil (orange bars) over TMTT (green bars); TMTT-M, male ACPs that preferred mineral oil (orange bars) over TMTT (green bars). The value in each bar represents the number of ACPs that made a choice. At least 60 ACPs were tested per treatment. Here, * indicates a significant difference between mineral oil and DMNT or TMTT at *P* < 0.05.

We then attempted to overexpress *CsCYP82L1* and *CsCYP82L2* in lemon leaves to assess their functions in defending against ACP. However, we could successfully overexpress only *CsCYP82L1* in lemon, which was confirmed by qRT–PCR (Fig. 7A). Therefore, we examined the preference of ACPs for leaves detached from WT and *CsCYP82L1*-overexpressing plants using dual-choice assay. Here, ACPs showed a lesser preference for transgenic lemon leaves than WT leaves (Fig. 7B). Earlier, RNA-seq and qRT–PCR had confirmed that homoterpene biosynthesis was activated in citrus infested by ACPs based on the upregulation of *CsNES* [(3*S*,6*E*)-nerolidol synthase] and *CsCYP82L1* (Fig. 3B; Supplementary Data Table S1). Besides, we found that ACP-infested citrus plants emitted more DMNT and TMTT than ACP-free plants (Fig. 1), and *CsCYP82L1*-OE callus produced DMNT using (*E*)-nerolidol as a substrate (Fig. 6B). Based on all these observations, we inferred that overexpressing *CsCYP82L1* in citrus plants resulted in the production of more DMNT using (*E*)-nerolidol as the substrate and reduced plant susceptibility to ACPs.

Further, to confirm the role of TMTT and DMNT, we investigated the behavioral response of ACPs to these volatiles using an airflow Y-tube olfactometer. Visual examination revealed that male ACPs preferred mineral oil over DMNT, and female ACPs preferred mineral oil over TMTT (Fig. 7C), suggesting that the two homoterpenes are almost unattractive to ACPs. Thus, we propose using these compounds to lower ACP infestation and HLB occurrence in citrus.

### CsERF017 binds to the promoter of *CsCYP82L1* and enhances its transcription

**Figure 8:**
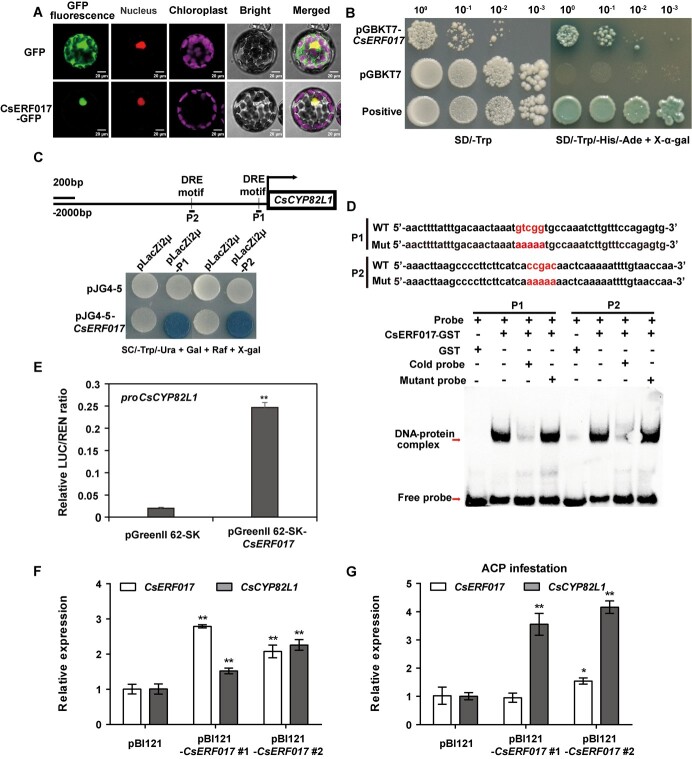
CsERF017 acts as a transcriptional regulator and directly activates *CsCYP82L1* by binding to its promoter. **A** Subcellular localization of CsERF017. 35S:CsERF017-GFP was introduced with pCAMBIA1300-NLS-mKATE containing a nuclear-localization signal (NLS) peptide into protoplasts isolated from *Arabidopsis* leaves; 35S:GFP served as the control. The fluorescence was visualized by confocal laser microscopy. Scale bars = 20 μm. CsERF017 was found localized in the *Arabidopsis* nucleus. **B** Trans-activation activity of CsERF017 in yeast. The ORF of *CsERF017* was fused to the GAL4 DNA-binding domain vector pGBKT7 and expressed in yeast cells. The transformants were then grown on SD/−Trp and SD/−Trp/−His/−Ade + X-α-gal medium. Yeast harboring the pGBKT7 vector was used as the negative control, and yeast harboring the pGBKT7-P53 plasmid was used as the positive control. **C** Y1H assay showing that CsERF017 binds to the truncated *CsCYP82L1* promoter (P1 and P2). The co-transformants were then grown on SC/−Trp/−Ura + galactose + raffinose + X-gal medium. In the *CsCYP82L1* promoter schematic, the black line represents the DRE core motif, and P1 and P2 represent truncated fragments containing the DRE core motif. **D** EMSA showing that CsERF017 binds to the DRE core motif in the *CsCYP82L1* promoter. Here, + and - denote the presence and absence of relevant probes or proteins, respectively. The 5′-ccgac-3′ is the motif bound by CsERF017 in ‘Probe’; this motif was replaced by 5′-aaaaa-3′ in the ‘Mutant probe’. **E** LUC/REN relative activity in tobacco leaves co-expressing *CsERF017* and *proCsCYP82L1*. Data shown are means ± standard deviation of three biological replicates. Here, ** indicates significant differences between WT and transgenic plants at *P* < 0.01. **F**, **G** Effect of transient overexpression of *CsERF017* on *CsCYP82L1* mRNA accumulation in lemon leaves before and after ACP infestation. Data shown are means ± standard deviation of three biological replicates. Here, * and ** indicate significant differences between the pBI121 empty vector-carrying plant and the *CsERF017* transgenic plant at *P* < 0.05 and *P* < 0.01, respectively.

To elucidate the upstream regulatory mechanisms of *CsCYP82L1*, its promoter was cloned and used to screen a Y1H cDNA library from *C. sinensis*. Only one gene, *CsERF017* (Cs4g07040), encoding 199 amino acids of a protein from the ERF transcription factor family, was identified (Supplementary Data Table S2). Multiple sequence alignment of CsERF017 with the homologous proteins from other plant species, including AtERF017 (*A. thaliana*), HuERF017 (*Herrania umbratica*), and CiERF017 (*Carya illinoinensis*), showed that all these proteins shared a typical AP2 domain. Moreover, CsERF017 was highly similar to AtERF017 (Supplementary Data Fig. S1). Further, to verify the subcellular localization of CsERF017, the CsERF017-GFP construct or the GFP control was co-transformed with a nucleus marker, NLS-mKATE, into *Arabidopsis* mesophyll protoplasts. Here, CsERF017 fused with GFP was co-localized with the nucleus marker, suggesting that CsERF017 is located in the nucleus and probably functions as a transcriptional regulator (Fig. 8A). Besides, yeast cells expressing BD-CsERF017 grew well on SD medium lacking Trp, His, and Ade but containing X-α-gal (Fig. 8B), suggesting that CsERF017 may be a transcriptional activator. Further *cis*-element analysis revealed that the *CsCYP82L1* promoter had two DRE core motifs (CCGAC) (Fig. 8C) and suggested that *CsCYP82L1* might be a downstream target of CsERF017. Subsequent Y1H assay demonstrated that only the Y2HGold yeast strain carrying pJG4-5-*CsERF017* with pLacZi2μ-P1 and pJG4-5-*CsERF017* with pLacZi2μ-P2 grew on SC/−Trp/−Ura + galactose + raffinose + X-gal medium, while other yeast co-transformants did not (Fig. 8C), suggesting that CsERF017 binds to the DRE core motif in the *CsCYP82L1* promoter. In EMSA, CsERF017 bound to the *CsCYP82L1* promoter but not to the mutant probe (Fig. 8D). Further, a dual luciferase assay was performed using constructs with the *CsERF017* ORF in pGreenII 62-SK vector and the *CsCYP82L1* promoter in pGreenII 0800-LUC vector. In this assay, *Nicotiana benthamiana* leaves co-injected with the *CsERF017* and *CsCYP82L1* promoters exhibited higher firefly luciferase (LUC) activity than the control (Fig. 8E). These observations confirmed that CsERF017 directly binds to the *CsCYP82L1* promoter (DRE core motif, CCGAC) and enhances its transcription.

Finally, *CsERF017* was transiently overexpressed in lemon leaves to assess the regulatory effect of CsERF017 on *CsCYP82L1*. As shown in Fig. 8F, overexpression of *CsERF017* in lemon leaves upregulated the expression of *CsCYP82L1* 5 days after infiltration. Moreover, the expression of *CsCYP82L1* in the *CsERF017*-overexpressing lines after ACP infestation was higher than that in the *CsERF017*-overexpressing lines without ACP infestation (Fig. 8F and G). These results indicated that CsERF017 could activate *CsCYP82L1* expression *in vivo.* Thus, the study suggests the generation of stable transgenic plants overexpressing *CsERF017* to regulate DMNT synthesis by increasing the mRNA accumulation of *CsCYP82L1* and thus repel ACP; however, this aspect needs to be investigated using further experiments.

## Discussion

Our previous study based on RNA-seq showed that two *CsCYP*s, *CsCYP82L1* (Cs5g27570) and *CsCYP82L2* (Cs5g27580), were significantly induced by ACP infestation in citrus leaves during the initial 48-h period (Supplementary Data Table S1). In this study, we first validated these expression levels using qRT–PCR (Fig. 3B and G). *CsCYP82L1* and *CsCYP82L2* in citrus infested by ACPs showed changes in expression patterns similar to the upregulation of *AtCYP82G1* in *A. thaliana*, *PtCYP79D*s in *P. trichocarpa*, *ZmCYP92C*s in *Z. mays*, and *CsCYP82D47* in *C. sinensis* following herbivory [2, 9, 15, 25]. These observations suggested that the overexpression of *CsCYP82L1* and *CsCYP82L2* in citrus leaves might contribute to the synthesis of homoterpenes in ACP-infested plants, subsequently acting against the insect pests or attracting the herbivore’s enemies.

Researchers have found that AtCYP82G1 forms TMTT from (*E*,*E*)-geranyllinalool and forms DMNT from (*E*)-nerolidol *in vitro*. However, expression of *AtCYP82G1* in the transgenic plants produced only TMTT, probably due to lack of the substrate required for (E)­nerolidol synthase activity [9]. Yeast recombinant expression and enzyme assays proved that both GhCYP82L1 and GhCYP82L2 convert (*E*)-nerolidol to DMNT and (*E*,*E*)-geranyllinalool to TMTT [12]. In this study, phylogenetic analysis showed that AtCYP82G1, CsCYP82L1, CsCYP82L2, GhCYP82L1, and GhCYP82L2 belonged to the same CYP82 family of dicot plants (Fig. 2B), suggesting similar catalytic roles for CsCYP82L1 and CsCYP82L2 in *C. sinensis*. Like AtCYP82G1, GhCYP82L1, and GhCYP82L2, both CsCYP82L1 and CsCYP82L2 catalyzed the *in vitro* formation of TMTT and DMNT from (*E*,*E*)-geranyllinalool and (*E*)-nerolidol, respectively (Fig. 5). However, in tea, which is also a dicot, recombinant CsCYP82D47 could transform (*E*)-nerolidol to DMNT but not (*E*,*E*)-geranyllinalool to TMTT [15]. Meanwhile, ZmCYP92C5 and ZmCYP92C6 of *Z. mays*, a monocot, formed a distant clade with AtCYP82G1. In an *Escherichia coli* system, ZmCYP92C6 specifically converted (*E*,*E*)-geranyllinalool to TMTT, while ZmCYP92C5 was capable of converting (*E*,*E*)-geranyllinalool to TMTT and (*E*)-nerolidol to DMNT [2]. These differences suggest the independent evolution of oxidative elimination in dicots and monocots. We also found that citrus callus overexpressing *CsCYP82L1* produced only DMNT, while those overexpressing *CsCYP82L2* made DMNT using (*E*)-nerolidol and/or TMTT using (*E*,*E*)-geranyllinalool (Fig. 6B, C, and F), suggesting differences in their *in vitro* enzymatic activities. This observation demands further studies on the mechanisms underlying the role and interplay between CsCYP82L1 and CsCYP82L2 in compartmentalizing terpene biosynthesis *in vivo*.

Typically, behavioral assays, coupled with identifying and characterizing semiochemicals, improve our understanding of insect communication and provide a basis to plan appropriate plant protection strategies [26]. Studies have confirmed that TMTT and DMNT are crucial in delivering direct defense (deterrents or repellents) against a broad spectrum of pests, such as aphids and leafhoppers [13, 14, 27]; they also act via the recruitment of natural enemies [9, 12, 18, 28–30]. For example, TMTT emitted by aphid-infested cotton repels aphids [27]. Rice plants that release TMTT and DMNT attract more natural enemies (*Cotesia chilonis*, a parasitoid) of the stem borer pest than normal ones [30]. Thus, identifying defense volatiles involved in ACP repellency will help manage ACP and breed ACP-resistant plants. An earlier study revealed that *A. occidentale* with high levels of DMNT and TMTT did not attract ACP like the ordinary hosts*.* Moreover, a man-made mixture of these two compounds inhibited the ACPs’ feeding behavior, suggesting they do not attract ACP [17]. Likewise, studies indicated that the constitutive release of high levels of DMNT from guava plants (*Psidium guajava* L.) is the major reason for lower HLB and ACP incidence in citrus under an intercropped system [31]. The present study assessed the influence of each chemical (TMTT or DMNT) on the preference of male and female ACPs*.* In the Y-tube assays, ACPs preferred the control arm containing mineral oil over the treatment arm containing TMTT or DMNT (Fig. 7C), indicating that either TMTT or DMNT reduces ACP attraction. These results suggest that TMTT and DMNT could be effective repellents of ACP.

Research during the last decade has demonstrated that altering plant volatile production by genetic and metabolic engineering could modify the behavioral response of pests. *TaPS* of wheat is a gene encoding a terpene synthase, and it specifically encodes a sesquiterpene synthase that catalyzes the conversion of *E*,*E*-farnesyl diphosphate to β-patchoulene. Overexpression of *TaPS* in *Arabidopsis* resulted in β-patchoulene production; these transgenics repelled beet armyworm (*Spodoptera exigua*) larvae [32]. Similarly, *OsTPS46* overexpression in rice increased (*E*)-β-farnesene and d-limonene emission, preventing attack by the aphid *Rhopalosiphum padi*. On the other hand, *OsTPS46* silencing made rice vulnerable to *R. padi*, which is not its usual pest [33]. Additionally, (*E*)-β-caryophyllene, a volatile sesquiterpene, from the transgenic sweet orange repelled ACPs [34]. Consistent with these reports, our study found that transgenic lemon overexpressing *CsCYP82L1* exhibited a decline in susceptibility to ACPs (Fig. 7B). These findings indicate the feasibility of improving the production of plant defense-associated volatiles through genetic and metabolic engineering.

Furthermore, numerous transcription factors have been reported to regulate the synthesis of plant secondary metabolites and directly affect the emission of volatiles associated with herbivore defense. For example, TPS10 in maize is mainly responsible for synthesizing (*E*)-β-farnesene and (*E*)-α-bergamotene in leaves damaged by *Ostrinia furnacalis* larvae. These two compounds attract *Cotesia marginiventris*, a parasitoid of spodopteran larval pests. Besides, the AP2/ERF family transcription factor EREB58 has been shown to regulate *TPS10* positively and stimulate (*E*)-α-bergamotene and (*E*)-β-farnesene emission*.* These two sesquiterpenes were accumulated in transgenic maize overexpressing *EREB58* but were undetectable in RNAi lines [35]. In tomato, SlMYB75 influences sesquiterpene accumulation by regulating the terpene synthase genes *SlTPS12*, *SlTPS31*, and *SlTPS35*. *SlMYB75* downregulation enhanced the accumulation of β-caryophyllene, α-humulene, and δ-elemene, improving plant tolerance to spider mites [36]. In this study, we found that CsERF017 promoted the expression of *CsCYP82L1 in vitro* and *in vivo* (Fig. 8), which could further influence DMNT accumulation. Thus, we propose regulating the expression of *CsCYP82L1* and increasing the release of homoterpene through *CsERF017* overexpression to repel ACPs.

## Materials and methods

### Experimental materials and design

Three-month-old ‘Succari’ seedlings with at least six fully expanded leaves (*C. sinensis* Osbeck) were maintained in a greenhouse under controlled conditions (28°C; 16 h light/8 h dark cycle, 65% relative humidity; 100 μmol m^−2^ s^−1^ light intensity) in this study. For the infestation assay, we first starved the *C*Las-free ACPs for 12 h and released them to cages with citrus plants (30 ACPs to each cage with four plants). The leaves were sampled for analysis before (control) and 12, 24, and 48 h after ACP infestation. For the exogenous hormone assay, leaves were sampled from citrus plants treated with SA (10 μmol l^−1^) or MeJA (100 μmol l^−1^) for differing durations (0, 0.5, 1, 3, 6, and 12 h). Then, to analyze the influence of mechanical damage, the citrus leaves were pierced with a microcapillary needle and sampled at 12-h intervals for 2 days. The stem, leaf, root, flower, peel, and pulp of these plants were also sampled at various stages, immediately frozen in liquid nitrogen, and stored in a −80°C freezer for subsequent RNA extraction to determine the tissue- and stage-specific expression patterns of various genes.

### Collection and identification of volatiles from citrus plants

One ACP-infested or ACP-free seedling with 20 leaves was kept in a glass jar (12 cm diameter; 32 cm height). After 3 h, the volatiles generated by the seedling were adsorbed onto a Tenax TA adsorbent (50 mg, 80/100 mesh, Shanghai ANPEL Scientific Instrument Company, China) by passing air (filtered using charcoal) at 300 ml min^−1^ for 3 h (11 a.m. to 2 p.m.) and analyzed by GC–MS. A GC–MS detection system (Agilent 8890/7000D) equipped with a SUPELCOWAX 10 column (Supelco Inc., 30 m × 0.25 mm × 0.25 μm) was adopted for the analysis, using helium as a carrier gas. The injector was maintained at 200°C for 1 min in splitless mode, and the carrier gas flow rate was set at 1.0 ml min^−1^. The GC temperature was initially set at 60°C for 3 min, and the oven temperature was increased to 240°C at a rate of 5°C min^−1^ and held for 15 min. Six replicates were analyzed per treatment.

### Identification and characterization of *CsCYP* genes

In this study, to identify the sequences of the putative CsCYPs that catalyze TMTT and DMNT formation in *C. sinensis*, a BLASTP search was conducted against the *C. sinensis* reference sequence using AtCYP82G1 (At3g25180), the CYP monooxygenase of *A. thaliana* [9]. Further, a sequence alignment was carried out with AtCYP82G1, and the putative CsCYPs were identified based on the conserved domains of the CYPs [37, 38], including PRD (PxxxxxxP), PD, HBD (FxxGxxxCxG), and SRS. Subsequently, the ClustalW program was used for the multiple sequence alignment of CsCYP82L and the CYPs from *A. thaliana*, *C. sinensis*, *G. hirsutum*, *O. sativa*, and *Z. mays*, and the MEGA-X program was used to build the phylogenetic tree with the maximum likelihood method and the Jones–Taylor–Thornton model (bootstrap analysis with 1000 replicates).

### Quantitative real-time PCR

Total RNA was extracted from different tissues (leaves, stem, root, flower, peel, and pulp) of WT citrus, treated leaves of WT citrus, and the calluses and leaves of transgenic plants with a FastPure Plant Total RNA Isolation Kit (Vazyme Biotech, China) and reverse-transcribed into cDNA using the PrimeScript™ RT Reagent Kit (+ gDNA Eraser; TaKaRa Bio, China). Gene expression was measured using this cDNA and a TB Green Premix Ex Taq™ II Kit (Tli RNaseH Plus; TaKaRa Bio, China) on a QuantStudio 5 Real-Time PCR Cycler (Applied Biosystems, NIST, USA), adopting annealing temperatures determined based on the gene-specific primers. The thermocycler program was set as follows: initial denaturation at 95°C for 30 s, followed by 40 cycles of amplification at 95°C for 5 s and 60°C for 34 s, and a final step of 95°C for 15 s, 60°C for 1 min, and 95°C for 15 s. The relative fold changes in the expression of the target genes were determined following the 2^−ΔΔCt^ method, using *CsActb* as the reference gene [39]. The *CsCYP82L1-* and *CsCYP82L2*-specific primers used in this assay are given in Supplementary Data Table S3. All assays were carried out using three independent biological replicates per sample.

### Subcellular localization of CsCYP82L1, CsCYP82L2, and CsERF017

The ORFs of *CsCYP82L1*, *CsCYP82L2*, and *CsERF017* without the termination codon were amplified with three separate pairs of primers (Supplementary Data Table S3; CsCYP82L1-GFP-F/CsCYP82L1-GFP-R, CsCYP82L2-GFP-F/CsCYP82L2-GFP-R, and CsERF017-GFP-F/CsERF017-GFP-R) and inserted into the pCAMBIA1300-GFP vector in-frame to the N-terminus of GFP to generate pCAMBIA1300-CsCYP82L1-GFP, pCAMBIA1300-CsCYP82L2-GFP, and pCAMBIA1300-CsERF017-GFP constructs. These plasmids were transiently expressed in *Arabidopsis* protoplast cells following a previously described method [40], using the plasmids pCAMBIA1300-sper-mKATE containing an ER membrane-localization signal peptide-encoding region and pCAMBIA1300-NLS-mKATE containing a nuclear-localization signal (NLS) peptide-encoding region for co-localization. The GFP and RFP signals were finally visualized under a fluorescence microscope (Nikon C2 system, Nikon Instruments, Japan).

### Heterologous expression in yeast and solid-phase microextraction coupled to GC–MS-based assay for CsCYP82L activity

The *CsCYP82L1* and *CsCYP82L2* ORFs were amplified with pYES2-CsCYP82L1-F/pYES2-CsCYP82L1-R and pYES2-CsCYP82L2-F/pYES2-CsCYP82L2-R primer pairs (Supplementary Data Table S3) and cloned into pYES2 vector. The generated recombinant construct was employed to transform yeast cells (*S. cerevisiae*; INVSc1 strain), and cells with the construct were selected on an SD medium lacking uracil and containing 2% glucose as the carbon source. Further, a single transformed colony was cultured in 3 ml of SD/−uracil liquid medium (30°C, 180 rpm, 24–48 h), and this starter culture was transferred to 50 ml of the same medium and allowed to grow until an OD_600_ of 0.6. The cell pellet obtained after centrifugation and rinsing with distilled water was used for subsequent assays.

For the enzyme activity assay, the transformed yeast cells (50 ml of SC/−uracil induction medium containing 2% galactose) were incubated at 30°C in a shaking incubator at 180 rpm for 12 h with exogenously added (*E*)-nerolidol (1 mM) or (*E*,*E*)-geranyllinalool (10 mM) (Yuanye Bio-Technology Co. Ltd, China). After 12 h, a SPME fiber assembly (polydimethylsiloxane fiber, 100 μm; Supelco) was quickly introduced into the headspace of the vial containing the cell mixture and maintained for 1 h at 30°C to adsorb the reaction products. Here, cells carrying the pYES2 empty vector were used as a control. The fiber was then inserted into a GC injector connected with an HP-5MS fused silica column (length, 30 m; thickness, 0.25 mm; film thickness, 0.25 μm; Agilent Technologies, Inc.) for 5 min to analyze the components. The GC oven was initially maintained at 50°C for 5 min; this temperature was then raised to 210°C at 3°C min^−1^ and further to 230 and 280°C at a rate of 10°C min^−1^. Meanwhile, the detector was kept at 250°C. Finally, TMTT (TRC Companies, Inc., Toronto, Canada) and DMNT (TRC Companies, Inc., Toronto, Canada) were identified using authentic substances.

### Generation of transgenic citrus callus overexpressing *CsCYP82L1* and *CsCYP82L2* and analysis of the metabolites

*Agrobacterium tumefaciens* (EHA105) cells were transformed separately with pCAMBIA1300-CsCYP82L1-GFP and pCAMBIA1300-CsCYP82L2-GFP plasmids. The positive colonies were used to transform the citrus callus (*C. sinensis* Osbeck cv. ‘Succari’) as described previously [41], and qRT–PCR confirmed the transgenic callus. Fresh citrus calluses obtained from the transgenic lines (*CsCYP82L1*-OE and *CsCYP82L2*-OE) were further grown on MT basal medium [42] with hygromycin B (25 mg l^−1^) and the WT callus was grown on MT basal medium for 7 days. One gram of the fresh callus was cultured in 35 ml of MT liquid medium with or without hygromycin B (25 mg l^−1^) containing malt extract (0.5 g l^−1^) and l-glutamine (1.5 g l^−1^) for another 7 days, followed by the addition of (*E*)-nerolidol (10 mM) or (*E*,*E*)-geranyllinalool (50 mM). After 72 h, the reaction products were adsorbed using SPME and examined via GC–MS, as mentioned above in the section Heterologous expression in yeast and solid-phase microextraction coupled to GC-MS-based assay for CsCYP82L activity. WT citrus callus was used as the negative control.

### Generation of *CsCYP82L1-*overexpressing transgenic lemon and analysis of ACP behavior

The ORF of *CsCYP82L1* was amplified using the pBI121-CsCYP82L1-F/pBI121-CsCYP82L1-R primer pairs (Supplementary Data Table S3) and cloned into pBI121. This pBI121-*CsCYP82L1* recombinant construct was introduced into *A. tumefaciens* (EHA105), and the positive colonies were cultured in LB broth containing kanamycin (50 mg l^−1^) and rifampicin (50 mg l^−1^) until an OD_600_ of 0.6–0.8. Lemon (*Citrus limon*) shoot segments were transformed with this culture following the *Agrobacterium*-mediated method [43], and the transformed explants were regenerated on MT medium containing 50 mg l^−1^ kanamycin to obtain the *CsCYP82L1*-overexpressing transgenic plant. Finally, qRT–PCR-validated plants showing *CsCYP82L1* expression were regarded as positive lines and used for further analysis.

A dual-choice assay was carried out using the control or *CsCYP82L1-*overexpressing plants to check the preference of ACPs, following a previously reported method [44]. First, one transgenic or WT leaf was kept on a water-soaked filter paper disc over 1.5% agar in a glass beaker (2 l). Then, 60 adult ACPs were released into each glass beaker after initial feeding and incubated at 26°C, 12-h photoperiod, and 65% relative humidity, and the ACPs on each leaf were counted after incubation for 4, 8, 12, 24, 48, and 72 h. Five beakers were maintained as replicates per treatment.

### Behavioral response trial

Behavioral bioassays were performed using a Y-tube olfactometer with an introduction arm (2 cm inner diameter and 15 cm length) and two lateral arms (15 cm length and 1 cm interior diameter) to evaluate the response of ACPs to TMTT and DMNT. Here, the two arms were connected to separate flasks. Each chemical (10 μl; 5 ng μl^−1^) dissolved in mineral oil was dropped onto a filter paper strip and placed in each flask, using the strip with mineral oil alone as the control in the other flask [45]. Then, charcoal-filtered air was pumped into each arm of the olfactometer at 0.2 l min^−1^.

Initially, the ACPs were released separately to the base of the Y-tube’s central arm and observed for 5 min. Psyllids that did not choose during this initial 5 min were removed and listed as having made no choice. Meanwhile, ACPs that moved to two-thirds of the length of the lateral arm and stayed there for at least 5 s were considered to have made a choice. During the assay, the Y-tube’s lateral arms were interchanged after each set of five turns to avoid position bias, and a fresh Y-tube was used after testing every 10 ACPs [46]. The bioassays were carried out between 10 a.m. and 4 p.m. and at least 60 female ACPs and 60 male ACPs were checked in one treatment.

### Trans-activation, yeast one-hybrid, electrophoretic mobility shift, and dual luciferase reporter assays

The *CsERF017* ORF was amplified with the pGBKT7-CsERF017-F/pGBKT7-CsERF017-R primer pairs (Supplementary Data Table S3) and cloned into the pGBKT7 vector to generate the pGBKT7-CsERF017 construct for the trans-activation assay. The Y2HGold yeast strain was then transformed with the plasmid and grown on SD/−Trp media at 30°C for 3 days, using the pGBKT7-P53 plasmid and the pGBKT7 empty vector as positive and negative controls, respectively. Further, all yeast transformants were cultured on SD/−Trp/−His/−Ade + X-α-gal media to examine trans-activation.

Two 50 bp *CsCYP82L1* promoter fragments (P1 and P2) containing the DRE core CCGAC were inserted into the pLacZi2μ vector to obtain the pLacZi2μ-P1 and pLacZi2μ-P2 constructs for the Y1H assay. Meanwhile, the *CsERF017* ORF was inserted into pJG4-5 to obtain the pJG4-5-CsERF017 construct. Then, yeast strain EGY48 was co-transformed with the fusion vectors and grown on SC/−Trp/−Ura + galactose + raffinose + X-gal media to examine reporter gene activation, using the empty pJG4-5 vector as the negative control. The primers are shown in Supplementary Data Table S3.

For EMSA, the GST-CsERF017 construct was generated by inserting the *CsERF017* ORF into pGEX-6p-3. Then, GST-CsERF017 protein expression was induced in *E. coli* BL21(DE3) cells by adding 0.5 mM IPTG, followed by incubation for 4 h at 37°C, and the expressed protein was purified from the cells using the GST-Tag Protein Purification Kit (Beyotime, China) as per the manufacturer’s guidelines. The *CsCYP82L1* promoter fragments (P1 and P2) containing the DRE core CCGAC and the mutated element were synthesized with biotin 5′-end-labeled oligonucleotides (Sangon Biotech, China). Finally, EMSA was carried out with a Chemiluminescent EMSA Kit (Beyotime, China) per the manufacturer’s guidelines, using the unlabeled oligonucleotides as competitors.

Further, a 2000-bp fragment of the *CsCYP82L1* promoter was amplified and inserted into pGreenII 0800-LUC to obtain the LUC reporter vector for LUC expression in the dual luciferase reporter assay. Similarly, the effector vector was generated by inserting the ORF of *CsERF017* into the pGreenII 62-SK vector with the CaMV35S promoter. These vectors were individually inserted into *A. tumefaciens* strain GV3101 (pSoup), and cells expressing the reporters and effectors were injected into tobacco leaves in different combinations, using the cells carrying the pGreenII 62-SK vector as the negative control. After 48 h, LUC and REN activities were detected with a Dual-Luciferase^®^ Reporter Assay System (Promega, USA) on a microplate reader (Molecular Devices, Switzerland). At least three biological replicates were maintained per combination. The primers are shown in Supplementary Data Table S3.

### Transient overexpression in lemon leaves

For transient expression in lemon leaves, the full-length coding sequence of *CsERF017* was amplified using the pBI121-CsERF017-F/pBI121-CsERF017-R (Supplementary Data Table S3) primer pairs and inserted into the pBI121 vector. The *A. tumefaciens* GV3101 cells containing pBI121-*CsERF017* or pBI121 were separately injected into the leaves of 4-week-old lemon plants, according to a previously described method [47] and incubated at 28°C for 5 days. The expression levels of *CsERF017* and *CsCYP82L1* before (control) and 24 h after ACP infestation were analyzed using qRT–PCR.

## Supplementary Material

Web_Material_uhae037

## Data Availability

All data generated during the study are provided in the published article and the supplementary files.
