# Supplementary material for: Identification and characterization of two P450 enzymes from Citrus sinensis involved in TMTT and DMNT biosyntheses and Asian citrus psyllid defense
Source: Hortic Res. 2024 Apr 1;11(4):uhae037. doi: 10.1093/hr/uhae037 (PMC11009467; doi:10.1093/hr/uhae037)
Supplement: Web_Material_uhae037 [file web_material_uhae037.zip › Figure S1.docx]

**Figure S1** Multiple amino acid sequence alignment of CsERF017 (Cs4g07040.1) with the homologous proteins of other plant species. AtERF017 (At1g19210.1, *Arabidopsis thaliana*), HuERF017 (XP_021276768.1, *Herrania* *umbratical*) and CiERF017 (XP_042972344.1, *Carya* *illinoinensis*). The sequence with the red underline represents the AP2 domain.
