# Supplementary material for: Identification and characterization of two P450 enzymes from Citrus sinensis involved in TMTT and DMNT biosyntheses and Asian citrus psyllid defense
Source: Hortic Res. 2024 Apr 1;11(4):uhae037. doi: 10.1093/hr/uhae037 (PMC11009467; doi:10.1093/hr/uhae037)
Supplement: Web_Material_uhae037 [file web_material_uhae037.zip › Table S2.docx]

| **Step** | **Procedure** |
| --- | --- |
| 1 | The promoter sequence of *CsCYP82L1* was cloned into the pAbAi vector to obtain the bait. |
| 2 | A bait/reporter system was generated by transforming the Y1HGold strain with the pbait-AbAi plasmid (*Pro-CsCYP82L1*-AbAi). |
| 3 | The Y1HGold bait strain was tested for background Aureobasidin A (AbA) expression. The minimal concentration of AbA for suppressing the basal expression of the Y1HGold bait strain (*Pro-CsCYP82L1*-AbAi) was 200 ng/mL (Fig. 1). Y1HGold strain transformed with p53-AbAi plasmid was used as a positive control.  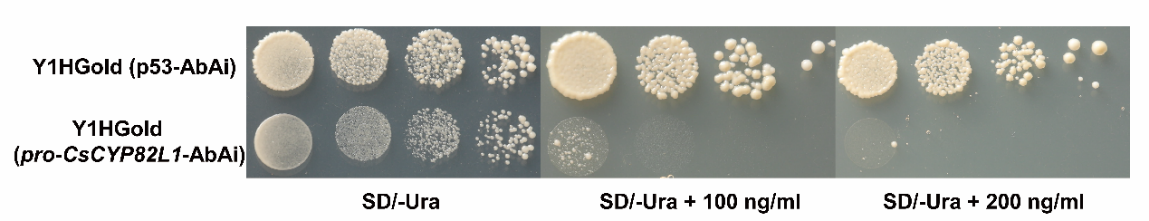  Fig. 1 Confirmation of minimal concentration of Aureobasidin A. |
| 4 | The pbait-AbAi plasmid (*Pro-CsCYP82L1*-AbAi) was co-transformed with a cDNA library into Y1HGold strain. The positive clones were identified by screening on the SD/-Leu/-Ura medium supplemented with AbA at 200 ng/mL (Fig. S2).  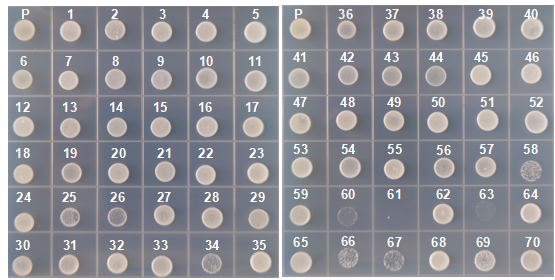Fig. 2 Screening and identification of the positive colonies. |
| 5 | Colony PCR, sequencing, and protein blast analysis confirmed the candidate gene. |

**Table S2.** Yeast-one hybrid (Y1H) screening of the upstream regulators of *CsCYP82L1*.
