# Supplementary material for: Identification and characterization of two P450 enzymes from Citrus sinensis involved in TMTT and DMNT biosyntheses and Asian citrus psyllid defense
Source: Hortic Res. 2024 Apr 1;11(4):uhae037. doi: 10.1093/hr/uhae037 (PMC11009467; doi:10.1093/hr/uhae037)
Supplement: Web_Material_uhae037 [file web_material_uhae037.zip › Table S3.docx]

**Table S3.** The list of primers used in this study.

| **Primers** | **Primer sequences (5'-3')** |
| --- | --- |
| qRT-CsCYP82L1-F | TGTGATGGGCGGACATACA |
| qRT-CsCYP82L1-R | CTTTCGGGTGGTTCAACAAC |
| qRT-CsCYP82L2-F | TTGCCGAAGAACCTGTTTG |
| qRT-CsCYP82L2-R | CCAACTGCTCACAATCAACG |
| CsCYP82L1-GFP-F | GGGGATCCTCTAGAGTCGACATGGATTCTACTTCTTTCAGC |
| CsCYP82L1-GFP-R | CTCACCATCTGCAGGTCGACAATGCATCGAAAAAGATCCT |
| CsCYP82L2-GFP-F | GGGGATCCTCTAGAGTCGACATGGATTTTATTTCTTTCAAGC |
| CsCYP82L2-GFP-R | CTCACCATCTGCAGGTCGACGAGGCGTTGATAAAGATCA |
| CsERF017-GFP-F | GGGGATCCTCTAGAGTCGACATGGTGAAGCACGTAGTC |
| CsERF017-GFP-R | CTCACCATCTGCAGGTCGACAAAATTCCAAAGAAACGAATCTTG |
| pGBKT7-CsERF017-F | CATGGAGGCCGAATTCATGGTGAAGCACGTAGTC |
| pGBKT7-CsERF017-R | GCAGGTCGACGGATCCTTAAAAATTCCAAAGAAACGAATCT |
| pYES2-CsCYP82L1-F | CTTGGTACCGAGCTCGGATCCACAATGGATTCTACTTCTTTCAGC |
| pYES2-CsCYP82L1-R | GCGGCCGTTACTAGTGGATCCTCAAATGCATCGAAAAAGATC |
| pYES2-CsCYP82L2-F | CTTGGTACCGAGCTCGGATCCACAATGGATTTTATTTCTTTCAAGC |
| pYES2-CsCYP82L2-R | GCGGCCGTTACTAGTGGATCCTCAGAGGCGTTGATAAAG |
| pBI121-CsCYP82L1-F | GAGAACACGGGGGACTCTAGAATGGATTCTACTTCTTTCAGC |
| pBI121-CsCYP82L1-R | CGATCGGGGAAATTCGAGCTCTCAAATGCATCGAAAAAGATC |
| Pro-CsCYP82L1-F | AGCTGATTATTTTTATAGCACAGCT |
| Pro-CsCYP82L1-R | GGTGATTTTGCCTGGGATTTG |
| pAbAi-pro-CsCYP82L1-F | AAATGATGAATTGAAAAGCTTAGCTGATTATTTTTATAGCACAGCT |
| pAbAi-pro-CsCYP82L1-R | AGCACATGCCTCGAGGTCGACGGTGATTTTGCCTGGGATTTG |
| pJG-CsERF017-F | GATTATGCCTCTCCCGAATTCATGGTGAAGCACGTAGTC |
| pJG-CsERF017-R | AGAAGTCCAAAGCTTCTCGAGTTAAAAATTCCAAAGAAACGAATCT |
| pLacZi2μ-P1-F | CAACTTTTATTTGACAACTAAATGTCGGTGCCAAATCTTGTTTCCAGAGTGG |

(continued)

| **Primers** | **Primer sequences (5'-3')** |
| --- | --- |
| pLacZi2μ-P1-R | TCGACCACTCTGGAAACAAGATTTGGCACCGACATTTAGTTGTCAAATAAAAGTTGGTAC |
| pLacZi2μ-P2-F | CAAACTTAAGCCCCTTCTTCATCACCGACAACTCAAAAATTTTGTAAC |
| pLacZi2μ-P2-R | CAAG  TCGACTTGGTTACAAAATTTTTGAGTTGTCGGTGATGAAGAAGGGGCTTAAGTTTGGTAC |
| pGEX-CsERF017-F | TTCCAGGGGCCCCTGGGATCCATGGTGAAGCACGTAGTC |
| pGEX-CsERF017-R | GATGCGGCCGCTCGAGTCGACTTAAAAATTCCAAAGAAACGAATCT |
| Probe-P1-F | AACTTTTATTTGACAACTAAATGTCGGTGCCAAATCTTGTTTCCAGAGTG |
| Probe-P1-R | CACTCTGGAAACAAGATTTGGCACCGACATTTAGTTGTCAAATAAAAGTT |
| Mutant-Probe-P1-F | AACTTTTATTTGACAACTAAATAAAAATGCCAAATCTTGTTTCCAGAGTG |
| Mutant-Probe-P1-R | CACTCTGGAAACAAGATTTGGCATTTTTATTTAGTTGTCAAATAAAAGTT |
| Probe-P2-F | AAACTTAAGCCCCTTCTTCATCACCGACAACTCAAAAATTTTGTAACCAA |
| Probe-P2-R | TTGGTTACAAAATTTTTGAGTTGTCGGTGATGAAGAAGGGGCTTAAGTTT |
| Mutant-Probe-P2-F | AAACTTAAGCCCCTTCTTCATCAAAAAAAACTCAAAAATTTTGTAACCAA |
| Mutant-Probe-P2-R | TTGGTTACAAAATTTTTGAGTTTTTTTTGATGAAGAAGGGGCTTAAGTTT |
| 62-SK-CsERF017-F | AGGACAGCCCAAGCTGAGCTCATGGTGAAGCACGTAGTC |
| 62-SK-CsERF017-R | GGGCCCCCCCTCGAGGTCGACTTAAAAATTCCAAAGAAACGAATCT |
| 0800-Pro-CsCYP82L1-F | CTTGATATCGAATTCCTGCAGAGCTGATTATTTTTATAGCACAGCT |
| 0800-Pro-CsCYP82L1-R | TGTTTTTGGCGTCTTCCATGGGGTGATTTTGCCTGGGATTTG |

(continued)

| **Primers** | **Primer sequences (5'-3')** |
| --- | --- |
| pBI121-CsERF017-F | GAGAACACGGGGGACATGGTGAAGCACGTAGTC |
| pBI121-CsERF017-R | CGATCGGGGAAATTCTTAAAAATTCCAAAGAAACGAATCT |
| *CsActb*-F | CCAAGCAGCATGAAGATCAA |
| *CsActb*-R | ATCTGCTGGAAGGTGCTGAG |
